# Epidemiology and Genetic Variability of HHV-8/KSHV among Rural Populations and Kaposi’s Sarcoma Patients in Gabon, Central Africa. Review of the Geographical Distribution of HHV-8 K1 Genotypes in Africa

**DOI:** 10.3390/v13020175

**Published:** 2021-01-25

**Authors:** Antony Idam Mamimandjiami, Augustin Mouinga-Ondémé, Jill-Léa Ramassamy, Délia Doreen Djuicy, Philippe V. Afonso, Antoine Mahé, Jean-Bernard Lekana-Douki, Olivier Cassar, Antoine Gessain

**Affiliations:** 1 Unité d’Epidémiologie et Physiopathologie des Virus Oncogènes, Institut Pasteur, CNRS, UMR3569, F-75015 Paris, France; mamimandjiami@gmail.com (A.I.M.); jill.lea.ramassamy@pasteur.fr (J.-L.R.); delia8691@yahoo.fr (D.D.D.); philippe.afonso@pasteur.fr (P.V.A.); 2 Unité des Infections Rétrovirales et Pathologies Associées, Centre International de Recherches Médicales de Franceville, Franceville BP-769, Gabon; ondeme@yahoo.fr; 3 Ecole Doctorale Régionale d’Afrique Centrale, Infectiologie Tropicale, Franceville BP-876, Gabon; lekana_jb@yahoo.fr; 4 Université de Paris, F-75006 Paris, France; 5 Centre Hospitalier Louis Pasteur, Service de Dermatologie, F-68024 Colmar, France; antoine.mahe@ch-colmar.fr; 6 Département de Parasitologie-Mycologie Médecine Tropicale, Faculté de Médecine, Université des Sciences de la Santé, Libreville BP-4009, Gabon; 7 Centre International de Recherches Médicales de Franceville, Unité de Parasitologie Médicale, Franceville BP-769, Gabon

**Keywords:** HHV-8, KSHV, epidemiology, serology, genetic variability, rural populations Gabon

## Abstract

Human herpesvirus 8 (HHV-8) is the etiological agent of all forms of Kaposi’s sarcoma (KS). K1 gene studies have identified five major molecular genotypes with geographical clustering. This study described the epidemiology of HHV-8 and its molecular diversity in Gabon among Bantu and Pygmy adult rural populations and KS patients. Plasma antibodies against latency-associated nuclear antigens (LANA) were searched by indirect immunofluorescence. Buffy coat DNA samples were subjected to polymerase chain reaction (PCR) to obtain a K1 gene fragment. We studied 1020 persons; 91% were Bantus and 9% Pygmies. HHV-8 seroprevalence was 48.3% and 36.5% at the 1:40 and 1:160 dilution thresholds, respectively, although the seroprevalence of HHV-8 is probably higher in Gabon. These seroprevalences did not differ by sex, age, ethnicity or province. The detection rate of HHV-8 K1 sequence was 2.6% by PCR. Most of the 31 HHV-8 strains belonged to the B genotype (24), while the remaining clustered within the A5 subgroup (6) and one belonged to the F genotype. Additionally, we reviewed the K1 molecular diversity of published HHV-8 strains in Africa. This study demonstrated a high seroprevalence of HHV-8 in rural adult populations in Gabon and the presence of genetically diverse strains with B, A and also F genotypes.

## 1. Introduction

Human herpesvirus-8 (HHV-8), also known as Kaposi’s sarcoma-associated herpesvirus (KSHV), was first identified in 1994 in a skin tumor biopsy from an AIDS-related Kaposi’s sarcoma [1]. This gammaherpesvirus is the etiological agent of Kaposi’s sarcoma (KS) [2,3,4] as well as primary effusion lymphoma [5] and most multicentric Castleman diseases and related lymphomas [6,7,8].

The geographical distribution of the virus is not ubiquitous. HHV-8 is indeed highly endemic in sub-Saharan Africa (particularly in central and eastern regions) and in the Mediterranean basin [9,10,11,12,13,14]. HHV-8 is also endemic in the northwestern part of China [15] and in Amerindian populations in South America [16,17,18,19]. HHV-8 prevalence is also high in subpopulations such as men having sex with men (MSM) [20,21,22,23]. The incidence of KS is elevated in HHV-8 endemic populations. 

Four main types of KS have been described: the classic, endemic, iatrogenic and the epidemic form [3,10,14]. The classic KS form is mainly present in the Mediterranean basin, while the endemic form is found in Africa and among Amerindian groups in South America. The epidemic type, which is currently the most common KS form, is associated with HIV infection; it was originally observed among MSM and is currently prevalent in many African countries. 

Saliva is the main mode of transmission for HHV-8 infection. In non-endemic countries, HHV-8 transmission occurs mainly during sexual intercourse [24], while in endemic areas, viral transmission appears to occur within families, mainly from mother to child and between siblings [13,25]. However, the situation may be more complex in some endemic areas and is not yet fully understood. Indeed, HHV-8 infection may be unevenly distributed from one region to another in some highly endemic areas (particularly in Africa), suggesting possible nonuniform specificities in the modes of transmission [26]. 

Studies on HHV-8 genetic variability have mostly focused on the K1 gene, which encodes a type I transmembrane protein that was previously found to have much greater sequence variability than the rest of the viral genome [27,28]. Indeed, K1 presents two highly variable regions (VR1 and VR2), and molecular epidemiology led to the definition of five main viral genotypes (A, B, C, D, E) [18,19,29,30,31,32,33,34,35,36,37,38,39,40,41] that cluster with the geographical origin of the infected individuals. Genotypes correlate neither with the nature of the HHV-8-associated disease (KS vs. PEL vs. MCD), nor with the type of KS. European and North African populations [27,28,42,43] as well as populations from Asia [31,44,45] are mainly infected with HHV-8 strains belonging to genotypes A1–4 and C. In contrast, genotype B and clade A5 of genotype A are predominant in sub-Saharan Africa [19,29,34,36,38,41,46,47,48]. Strains of genotype D mainly infect individuals living in or originating from the Pacific region [30,32,49], while E genotype has been reported exclusively among Amerindians [16,18,33].

Although located in a highly endemic area, there is a lack of knowledge on the epidemiology of HHV-8 and KS in Gabon [50,51]. The present work aimed to gain new insights into the epidemiology of HHV-8 and its genetic diversity in this central African country.

## 2. Materials and Methods 

### 2.1. Rural Population Survey

This study focused on a rural subgroup of the population sampled in Gabon and described earlier [52]. Briefly, the initial study was conducted between 2013 and 2017 in rural villages and settlements of Bantu and Pygmy populations. A systematic enrolment strategy was carried out and 1020 volunteers over the age of fifteen were included. A 5–10 mL whole blood sample was collected in EDTA vacuum tubes from all consenting individuals who met the inclusion criteria. Plasma and buffy coat were obtained 48–72 h after collection and stored frozen at −80 °C. DNA extracted from five individuals, four men and one woman (mean age 43 years), with Kaposi’s sarcoma tumors were also included in the study.

### 2.2. Ethical Statement

The ethical approval was given by the National Ethics Committee of Gabon under the approval number 00021/2017/SG/CNE issued on 25 May 2017. Prior to inclusion, written informed consent from the community and individuals was sought and obtained from participants after detailed information about the study was provided. Children over 15 years of age were included after they provided their assent and with informed consent of their legal tutor. 

### 2.3. HHV-8 Serological Tests

Detection of anti-LANA (latency-associated nuclear antigen) antibodies was performed on 1020 plasma samples by an indirect immunofluorescence assay (IFA) using a BC-3 cell line, as previously described [30]. These cells, derived from primary effusion lymphoma, were positive for HHV-8 and negative for EBV [53]. They expressed only the latency-associated nuclear antigen (LANA). Briefly, BC-3 cells in the exponential growth phase were first washed and suspended in PBS. Twenty thousand cells were coated on each well of the slide, then dried and fixed by immersion in glacial acetone. The slides could be stored at −80 °C for later or immediate use. In this case, the cells were incubated with 25 μL of the tested plasmas diluted in PBS for 1 h at 37 °C. For each slide, one well was used as the HHV-8 positive control. One well per set of slides was also dedicated to the negative control. The slides were then washed with PBS for 15 min. After incubation for 1 h at 37 °C with 25 μL of a 1/100 diluted solution of Evans blue and fluorescein-coupled anti-human immunoglobulin G antibodies, the slides were washed again in PBS for 1 h. The assembly between slide and cover slide was carried out with Vectashield. The slides were then observed using an Aristoplan microscope with LED light source. The determination of the antibody titer, anti-LANA, of each sample was evaluated by serial dilutions of the plasma (1:40, 1:80, 1:160). The positivity was defined by the presence of dotted nuclear reactivity at each dilution. The results of each dilution were interpreted by three independent experienced users. A sample was considered positive at a given dilution when at least two observers had identified it as positive.

### 2.4. Molecular and Phylogenetic Analyses of HHV-8

High molecular weight DNA was extracted from frozen peripheral blood leucocytes (buffy coat) of all 1020 participants using the QIAmp DNA blood mini kit (Qiagen, Hilden, Germany) or from KS frozen biopsies using the QIAmp DNA mini kit (Qiagen, Hilden, Germany). After quantification, the extracted DNA was subjected to polymerase chain reaction (PCR) amplification. The human β-globin gene was first targeted to assess the amplifiability of the DNA. Then, HHV-8 infection was determined by nested PCR to obtain a 737-nt long fragment of the K1 gene (ORF-K1) using K1AG75s/K1AG1200 [38] and VR1s/VR2as1 [54] primer sets. Following electrophoresis, PCR products were purified and sequenced on both strands. The ClustalW algorithm in MacVector 6.5 software (Oxford Molecular, Hunt Valley, MD, USA) was implemented to align forward and reverse sequences of each segment to obtain a consensus sequence of the full K1 gene.

### 2.5. Nucleotide Sequence Accession Numbers

The 31 new nucleotide sequences were all deposited in GenBank under accession numbers MT900801 to MT900831.

### 2.6. Statistical Analyses

Seroprevalences obtained at each dilution and the detection rate of HHV-8 infection, determined by PCR, were calculated with their 95% confidence intervals (95% CI). Univariate analysis was performed accordingly using chi-squared tests or chi-squared tests for trends to compare HHV-8 seroprevalence and percentage of PCR positive rates between sex, ethnic group, age groups and among the different provinces. Age was compared between Pygmy and Bantu ethnic groups using Student’s *t*-test. All analyses were performed using STATA 15.0 software (Stata Corporation, College Station, TX, USA).

### 2.7. HHV-8 Genotype Distribution: Search Strategy and Data Extraction

We conducted a review of published articles from African countries on HHV-8 genotypes. All study types (case reports, retrospective, prospective and observational) were included whenever the data from these studies analyzed the full or partial K1 gene. Electronic search databases such as Medline and Web of Science were analyzed for relevant data published between 1999 and 2020. The search terms used were: (“K1” OR “K1 gene” OR “genotype” OR “genotypic” OR “genotype distribution” OR “genetic variability” OR “genetic diversity” OR “genetic polymorphism” OR “genetic characterization” OR “molecular epidemiology” OR “phylogenetic analysis” OR “sequence analysis”) AND (“HHV8” OR “HHV-8” OR “KSHV” OR “herpesvirus 8, human” OR “human herpesvirus 8” OR “Kaposi sarcoma associated herpesvirus” OR “Kaposi’s sarcoma associated herpesvirus” OR “Kaposi sarcoma-associated herpesvirus” OR “Kaposi’s sarcoma-associated herpesvirus”). All these query items were associated with the name of a different African country. From the aforementioned search options, two reviewers downloaded the relevant articles and assigned each article using a ranking grid. These ranks were based on country of study, study objectives, study population, sampling size, source of DNA, genetic characterization of HHV-8 for K1 gene and the size of K1 gene analyzed. 

## 3. Results

### 3.1. Rural Populations

We studied samples from 1020 people (632 men and 388 women) living in rural areas in five provinces of Gabon (Table 1) [52]. The studied population consisted mainly of Bantus (931/1020; 91%) and few Pygmies (89/1020; 9%). The mean age of the studied population was 51.2 (range, 15–95 years). The Pygmy subgroup was significantly younger than the Bantu subgroup (mean ages were 40.9 and 52.1 years, respectively, Student’s *t*-test *p*-value < 0.001).

### 3.2. Sero-Epidemiology of HHV-8 in Rural Populations

**Figure 1:**
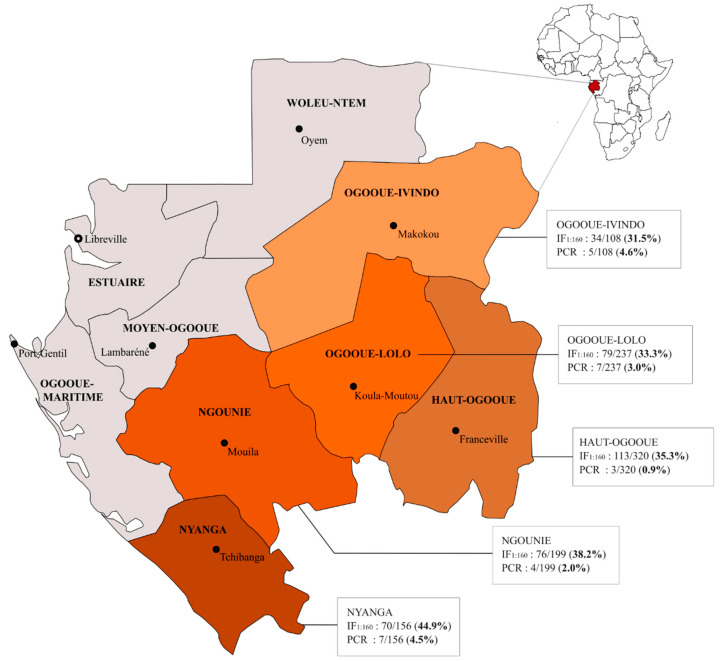
HHV-8 serological and molecular results in five studied provinces in Gabon.

The 1020 plasmas were tested by IFA at three dilutions (1:40, 1:80 and 1:160). As expected, the number of seropositive samples decreased with the dilution factor: 493 at the 1:40 (48.3%), 455 at the 1:80 (44.6%) and 372 at the 1/160 (36.5%) (Table 2) (chi-squared test for trend *p*-value < 0.001). To reduce the risk of false positive results, we considered the 1:160 dilution, which gave an overall HHV-8 seroprevalence of 36.5% (95% CI 33.5–39.5%) in the study population. Seroprevalence rates of HHV-8 were comparable between sex (36.2% of male and 36.9% of female, *p*-value = 0.84) and were not associated with age (*p*-value = 0.65) (Table 1). In addition, there was no significant difference in HHV-8 seroprevalence among ethnic groups (36.8% for the Bantus and 33.7% for the Pygmies, *p*-value = 0.57). This rural population was previously tested for HTLV-1 infection [52] and the prevalence of HTLV-1 was 12.2% in this subgroup (124/1020; 95% CI 10.2–14.3%). There was no significant difference in HHV-8 seroprevalence by HTLV-1 status. Thus, HHV-8 seroprevalence was 37.2% among HTLV-1 negative individuals (333/895) and 31.5% among HTLV-1 positive individuals (39/124) (*p*-value = 0.22). Finally, HHV-8 seroprevalence ranged from 31.5% in the Northeast (Ogooué-Ivindo province) to 44.9% in the South (Nyanga province), but the differences were not statistically significant (*p*-value = 0.12) (Figure 1 and Table 1). Of note, similar conclusions were achieved when using seroprevalences at different dilutions (i.e., 1:40 and 1:80).

For each province, the data provided indicate the number of HHV-8 positive individuals among the number of individuals tested and obtained by serological (IFA at the 1:160 dilution) and molecular technique (PCR) and the resulting seroprevalence and percentage of positive PCR, respectively. This map was modified from https://commons.wikimedia.org/wiki/Atlas_of_Gabon using Inkscape 0.92.2.

### 3.3. HHV-8 Molecular Results

Genomic DNA was extracted from buffy coats for the 1020 tested individuals. All samples were amplifiable for the human β-globin gene. When subjected to HHV-8 K1 PCR, 35 samples generated an amplicon at the expected size, of which 26 could be sequenced directly and corresponded to the ORF-K1 gene (Table 2).

The others gave no sequence reaction or were nonspecific. Thus, the global detection rate of HHV-8 infection (as detected by PCR) was 2.6% (26/1020; 95% CI 1.7–3.7%). The percentage of K1 positive PCR did not vary with gender (2.5% of men vs. 2.6% of women, *p*-value = 0.96) and did not increase with age (*p*-value = 0.44) (Table 1). 

HHV-8 DNA was detected in 3.5% of seropositive individuals at dilution 1:160 (13/372) and in 2.0% of seronegative individuals (13/648). 

The percentage of HHV-8 positive PCR was not significantly different according to the serological status at this dilution (*p*-value = 0.15). However, the number of HHV-8 PCR positive individuals was significantly higher when we considered seropositivity at dilutions of 1:40 (*p*-value = 0.01) and 1:80 (*p*-value = 0.03). This difference can be explained as anti-HHV-8 antibodies from some infected individuals were only detected at lower dilution. Indeed, of the 26 individuals with a positive PCR, 19 had antibodies detected at the dilution of 1:40; 17 at 1:80 and only 13 at 1:160. HHV-8 PCR detection rate was therefore 3.9% (19/493; 95%CI 2.3–6.0) and 3.7% (17/455; 95%CI 2.2–5.9), respectively, in individuals who were seropositive for the 1:40 and 1:80 dilutions (Table 2).

### 3.4. HHV-8 Genetic Variability

In addition to the 26 sequences obtained from apparently healthy individuals, we generated 5 sequences from biopsies obtained from patients with epidemic KS. Overall, we obtained 31 HHV-8 sequences from Gabon.

The alignment of the 31 sequences showed that 29 sequences were distinct. In two occasions, the samples were identical in a couple (Gab045NY/Gab111NY and Gab185OL/Gab189OL). 

Pairwise comparison of the 29 different sequences revealed a nucleotide polymorphism of 17.9% and a polymorphism in amino acids reaching 34.1%. This is consistent with the fact that the K1 protein is the target of the immune system and is prone to nonsynonymous mutation accumulation [55]. After alignment with reference strains belonging to genotypes A and B commonly found in Central Africa, 24 strains belonged to the B genotype and 6 to the A genotype. The last one (Gab135NY) was closely related to the few known strains belonging to the F genotype. In particular, this Gab135NY strain was only 6.5% different from the Uganda F genotype strain (HKS22) in nucleotides and 10.9% in amino acids.

The 22 unique genotype B sequences exhibited a 0.1% to 8.7% nucleotide divergence, while the five unique A5 sequences showed a divergence of 0.1% to 2.2%. Amino acids polymorphism reached 14% and 6.5% for the B and A5 genotypes, respectively. 

### 3.5. HHV-8 Phylogenetic Analyses

**Figure 2:**
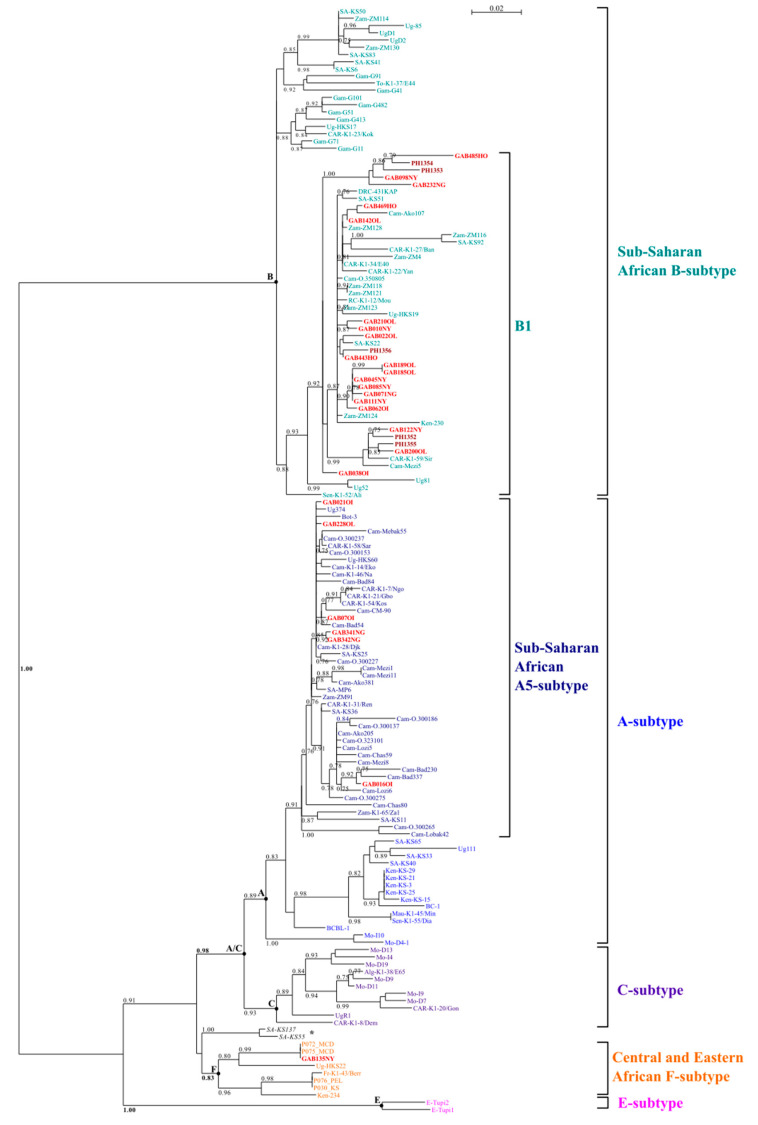
Phylogenetic analysis of the 31 new Kaposi’s sarcoma-associated herpesvirus (KSHV)/HHV-8 sequences. Phylogenetic comparisons were performed with the 689 nucleotide long K1 gene fragment obtained from 153 KSHV/HHV-8 isolates, including the 31 sequences generated in this study (in red) and 122 previously published sequences. The phylogeny was derived by the maximum likelihood (ML) method with the GTR model. Horizontal branch lengths are drawn to scale, with the bar indicating 0.02 nucleotide replacements per site. The maximum posterior probabilities were calculated and reported on the branches with threshold value ≥0.75. * Two strains characterized from South Africa (KS55 and KS137) are not yet molecularly classified [34].

The initial phylogenetic analyses were carried out on 689 bp long sequences, including the 31 new strains, as well as 122 K1 prototype sequences (Figure 2). These 122 HHV-8 strains included many of the sequences characterized for the complete or near complete K1 gene in individuals from African countries, plus most of the classical prototype strains such as BC-1 (AF178807) and BCBL-1 (JN800483) for the A genotype and Ug52 (AF130290) and Ug81 (AF130291) for the B one. For the African strains, we carefully selected the strains from 14 different countries (Algeria, Botswana, Cameroon, Central African Republic, Democratic Republic of Congo, Gambia, Kenya, Mauritania, Morocco, Senegal, South Africa, Togo, Uganda and Zambia), for which some K1 sequences were available. For countries with a high genetic diversity, such as South Africa, we included several strains in the analysis. In addition, we also included all of the most variant strains, such as, for example, the F genotype strains from Uganda (HKS22-AY953882) and Kenya (KE-234-FJ884616). The Amerindian E genotype was used as outgroup (Tupi-1-AF220292 and Tupi-2-AF220293). The analyses were based on two different phylogenetic methods (neighbor joining and maximum likelihood), which gave similar phylogenetic topologies.

Most of the strains (24/31 = 77%), including the 5 KS strains, belonged to the B genotype clade, more precisely the B1 subgroup. This clade contains sequences previously characterized from Cameroon, Central African Republic and from Democratic Republic of Congo Kenya, South Africa, Uganda and Zambia. The other sequences (6/31 = 19%) clustered within the A5 genotype clade, with sequences originating from various countries from Central and Austral Africa (South Africa and Botswana). There was no segregation of viral genotypes according to localization in Gabon: B1 and A5 subgroups were found in different provinces. Finally, the Gab135NY strain clustered in the genotype F clade, which was previously found in Kenya (KE-234-FJ884616), Uganda (HKS22-AY953882) and in France among men having sex with men (MSM) (K1-43/Berr-AF178810; P072_MCD-MK876734; P075_MCD-MK876735; P076_PEL-MK876736; P030_KS-MK876732).

### 3.6. Review of the Diversity of HHV-8 Strains in Africa Based on the K1 Gene Study

**Figure 3:**
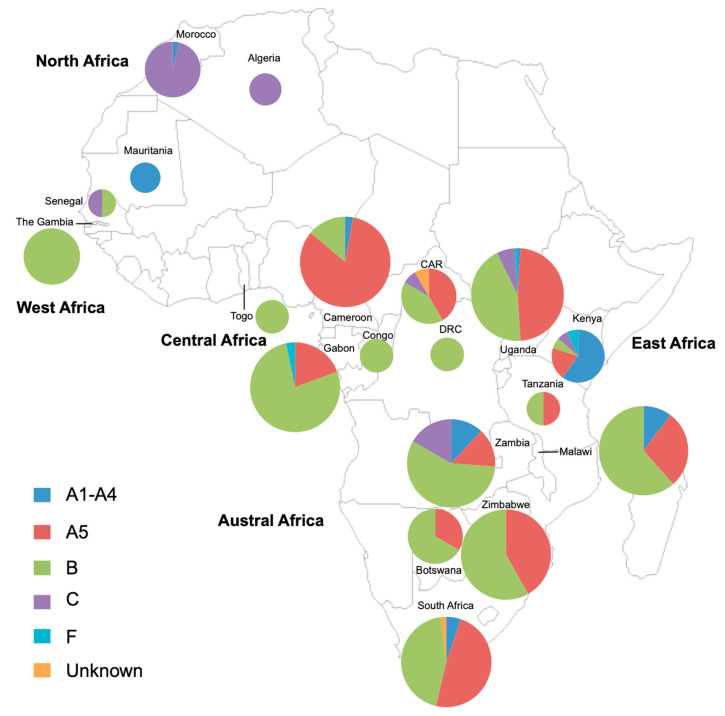
Map of Africa indicating the distribution of HHV-8 genotypes.

Four hundred and eighty-three HHV-8 strains for which the K1 region (complete or partial) was available were found from our review research and were classified according to the country of origin of the infected persons. These strains originated from 19 different African countries (34%; 19/54). Genotypes A1–4, A5, B, C, F and unidentified HHV-8 genotypes were found at frequencies of 5.3% (26/483), 38.1% (184/483), 46.3% (224/483), 9.1% (44/483), 0.6% (3/483) and 0.6% (3/483), respectively. These data were then used to map the genotypic distribution of HHV-8 strains by country (Figure 3).

Figure 3 shows the distribution of HHV-8 genotypes in 19 African countries based on K1 gene analysis and including the analysis of 483 viral strains: 13 strains from North Africa (Algeria/Mauritania/Morocco), 12 strains from West Africa (Senegal/The Gambia/Togo), 80 strains from Central Africa (Cameroon/Central African Republic (CAR)/Congo/Democratic Republic of Congo (DRC)/Gabon), 113 strains from East Africa (Kenya/Tanzania/Uganda) and 248 strains from Austral Africa (Botswana/Malawi/South Africa/Zambia/Zimbabwe). Countries without any indication have not yet published data on HHV-8 genotypes to our knowledge. The size of the circles is proportional to the number of HHV-8 strains characterized: The smallest size is for less than 5 strains; the intermediate size is for 6 to 30 strains and the largest size is for a number of strains greater than or equal to 31.

## 4. Discussion

This work was the first large-scale study carried out on HHV-8 in Gabon, a Central African country. It was conducted on a large population (1020 adults from rural areas) scattered throughout the country and combined a sero-epidemiological study (based on the detection of plasma anti-LANA antibodies) with molecular aspects (aimed at characterizing the viral genotype). We found that 36% of the tested population was sero-reactive to LANA at a 1:160 dilution, and 2.6% were positive to PCR, with most strains belonging to genotype B and at a lesser extent to genotypes A5 and F.

The high HHV-8 seroprevalence we observed in Gabon did not vary according to age, sex or ethnic group and was similar in the different studied localities. This confirms and extends to a larger population more representative of Gabon a preliminary work that was focused on a targeted population of 344 pregnant women (mean age 24 years) in the Lambarene region [50]. Indeed, in this specific group, HHV-8 seroprevalence was 31% using an IFA test (for the detection of anti-latent antibodies) at a dilution of 1:20, with no variation according to age [50]. Moreover, our results are close to those obtained with a similar serological method for a comparable population (large rural Bantu group, living in southern Cameroon close to the northern border of Gabon) [29]. However, this study found an increase of HHV-8 seroprevalence with age, particularly significant in the Pygmy group from Cameroon. Other studies, especially in adult populations in Africa or of African origin, showed higher levels of seroprevalence (about 50 to 90%) with or without an increase with age [56,57,58,59,60]. These disparities can be explained in part by the different serological methods and their sensitivity (e.g., IFA vs. recombinant ELISA and peptide EIA) or by the different antigenic targets (latent vs. lytic antigens) and differences in the cut-off thresholds used for each assay (here we considered 1:160 dilution). The differences could also likely reflect the various studied populations (urban vs. rural, Pygmies vs. Bantus, etc.) and the different epidemiological designs [12,29,61,62].

The detection of anti-latent antibodies is generally considered to be more specific than the detection of anti-lytic antibodies [3,12,63,64]. This implies that the seroprevalence of HHV-8 is generally lower using an anti-latent test. This was well-illustrated by a hospital study in northern Cameroon, which used both IFA tests (one anti-latent and one anti-lytic). In this work, carried out on nearly 300 children and adults, the seroprevalence of anti-lytic antibodies was twice as high (51%) as that of latent antigens (25%) [62]. Similarly, in a patient population with epidemic KS in the Central African Republic, the detection of anti-LANA antibodies was less frequent, being present only among 24 of 34 (71%) plasma samples tested, than the detection of anti-lytic ones (94%). Moreover, the geometric mean of antibody titers against HHV-8 lytic antigens was higher than for anti-LANA antibody titers [65]. These data may suggest that the expected overall HHV-8 seroprevalence in Gabon is probably higher than observed in our study. There was no significant difference in HHV-8 seroprevalence between Bantus and Pygmies in our study, in contrast to the study by Betsem et al., which found a lower seroprevalence rate in Pygmies from South Cameroon [29]. Though this lack of difference can mainly be explained by the low number of Pygmies included in our study, further studies in larger groups are in progress to better appreciate the HHV-8 epidemiological situation in the different Pygmies groups living in Gabon and Cameroon and to decipher some socio-environmental and/or biological characteristics (environmental cofactors, specificities of lifestyles influencing modes of transmission or genetic factors) that may contribute to possible differences between Bantus and Pygmies.

In highly endemic general populations, particularly in Africa and South America, several studies have shown that HHV-8 acquisition occurs primarily in children, with transmission between siblings and from mother-to-child, while transmission between adults appears to be less frequent [60,66,67,68,69]. The question of why HHV-8 transmission is mainly limited in the general population to these specific age groups remains unanswered (for review see [26,56,70]). Regardless of the population studied and the socio-cultural practices used, transmission is currently considered to be primarily through HHV-8 infected saliva [24,57]. Of note, HHV-8 DNA is detected more frequently and at higher levels in saliva than in blood buffy coat [71].

In this study, we detected HHV-8 in buffy coat DNAs in 2.6% of the total population tested and in 3.5% and 3.9% of the HHV-8 seropositive individuals at dilution of 1:160 and 1:40, respectively. This is rather lower than what has been found in other epidemiological studies. In fact, in research carried out on relatively large and predominantly adult populations in Cameroon, Vanuatu and Siberia, the detection rates by nested PCR varied from 5% to 20% of HHV-8 seropositive individuals [29,30,31]. These differences can be related to the origin and quality of DNA samples (peripheral blood mononuclear cells vs. buffy coat), the origin of the populations tested, and especially, as seen above, to the serological method used, which can strongly vary the seroprevalence of HHV-8.

From a molecular point of view, one strain of our series belonged to the rare F genotype. This variant was first identified in 2000 in a French homosexual male patient of Caucasian type suffering from HIV-associated PEL with ascites (K1–43/Berr). At that time, we described this strain as a distinct variant of the known genotypes, with arguments for independent evolution [38]. Such variant was considered in 2006 as a new genotype (labelled F) by Kajumbula et al., who reported a second related strain in a patient from Uganda [35]. Interestingly, a recent report described the presence of such variants in 4.5% of Caucasian MSM living in Paris, France, but not in other HHV-8 infected epidemiological groups of the same area [72]. The remaining strains we described belonged either to the B1 or the A5 genotypes, typical from the sub-Saharan African regions. In Gabon’s neighboring countries, the ratio between B1 and A5 strains varies: in Cameroon the majority of strains were A5 (27/32; 84%) [29,38], in contrast to the current study where the majority were B1 strains (24/31; 77%). In the Central African Republic, where only 12 HHV-8 K1 strains are available to date (from AIDS-KS), the A5 and B strains are equally distributed [38].

We have seen above that based on the variability of the K1 region, the different HHV-8 genotypes present in the world (A, B, C, D, E, F) are clearly related to the geographical origin of infected patients. Furthermore, in some cases, such as genotype D, endemic in the Pacific region, there is a well-defined, geographically linked intragenotyic diversity, probably reflecting past movements of populations infected with these molecular variants. In addition, a founder effect, followed by local genetic drift of viruses, may also have played a role in this genotypic restriction. Indeed, although belonging to the widely distributed clade D, the Japanese D strains are different from those from Taiwan, which are different from those from Vanuatu, also different from those of Polynesian origin [30]. It is therefore questionable whether such a geographical restriction may exist among HHV-8 genotypes present on the African continent, the largest HHV-8 endemic area. As an example, such a viral restriction has been well demonstrated in West and North Africa for the cosmopolitan genotype of HTLV-1 [73]. To try to answer this question, we have listed and analyzed almost all HHV-8 viral strains of African origin for which the K1 region (or a fragment of it) was available in the databases. Although there is a clear lack of data for many countries from North and West Africa, the following main conclusions can be drawn on the basis of these still very preliminary data: (1) The majority of the viral strains, present in sub-Saharan Africa, belong primarily to clade B and secondly to A5; indeed, if we consider the 7 countries (Cameroon, Gabon, South Africa, Malawi, Uganda, Zambia and Zimbabwe) for which more than 30 strains have been analyzed, in 4 cases (Gabon, Malawi, Zambia, Zimbabwe) the genotype B is in the majority, ranging from 57 to 77% of all strains; (2) The genotype A5 represents around 38% of the strains, ranging from 14% to 42%; (3) In North Africa, (at least in Morocco) genotype C is the predominant clade [42]; (4) Other genotypes are much rarer, represented by some F strains present, e.g., in Kenya [41] and Uganda [35] and other strains not yet classified and present for example in South Africa [34]; (5) The question of whether within genotype B or A5 there is genetic variability that could be limited to a specific and given geographical area remains open. It is unclear why such subgroups are not easily identifiable. We can postulate that in West and Central Africa there is no clear genetic isolation of populations that would lead to local speciation. In this region, genetic intermixing is likely to be frequent. However, to demonstrate such specific viral clustering, an analysis of a much larger number of strains of varied origin on a large fragment of the K1 gene (ideally the complete one) is necessary. 

The origins of the A5 genotype remain obscure: How can a strictly sub-Saharan clade have emerged within the A/C genotype, which is typically related to Caucasian or Asian populations. It is clear that speciation between A1–4 and A5 is recent. Indeed, when considering the non-variable region of the K1 gene, only few mutations have been accumulated, and A1–4 and A5 are undistinguishable [29]. Zong et al. proposed that such diversification could be as recent as 4000 years ago [74]. As the A5 subtype seems to be at the roots of the A genotype, we initially proposed that the genotype A was primarily African and that A1–4 diversified in response to immune pressure in Caucasian and Asian populations [29]. In sharp contrast, others have proposed that instead the A/C genotype had spread in Eurasia, and A5 was the consequence of a re-introduction of the virus in Africa, followed by a very rapid propagation of the virus (coinciding with the expansion of the Bantu people) [48,74]. 

To better understand the origins and evolution of HHV-8, future studies should require the sequencing of several genomic regions—or the entirety of the genome—of a series of strains originating from different geographical areas. Indeed, it has been recently demonstrated that recombination might be a major feature of HHV-8 evolution. We and others initially identified recombination within the K1 gene, especially in the C type, either intertypic or intratypic [27,42,74]. Since then, multiple recombination breakpoints have been identified throughout the genome [75]. This explains why subgroups defined on K1 and K15 do not always cluster. Identifying and dating the recombination events will shine new light on the origin and the evolution of the different viral genotypes and will lead to a better description of the migration and intermixing of infected populations.

## Figures and Tables

**Table 1:** Human herpesvirus-8 (HHV-8) serological (IFA) and molecular (PCR) results.

	*n*	HHV-8 Serological Results (IFA)			HHV-8 Molecular Results (PCR)		
		*n*IFA 1:160	Seroprevalence (95%CI)	*p*-Value	*n*PCR K1	Percentage of Positive PCR (95%CI)	*p*-Value
Sex							
Female	388	143	36.9% (32.0–41.9)	0.84	10	2.6% (1.2–4.7)	0.96
Male	632	229	36.2% (32.5–40.1)		16	2.5% (1.5–4.1)	
Age category							
15–35	238	85	35.7% (29.6–42.2)	0.65	4	1.7% (0.5–4.2)	0.44
36–50	250	86	34.4% (28.5–40.6)		8	3.2% (1.4–6.2)	
51–65	276	109	39.5% (33.7–45.5)		5	1.8% (0.6–4.2)	
66–95	256	92	35.9% (30.1–42.1)		9	3.5% (1.6–6.6)	
Ethnic group							
Bantus	931	342	36.8% (33.6–39.9)	0.57	26	2.8% (1.8–4.1)	-
Pygmies	89	30	33.7% (24.0–44.5)		0	-	
Province							
Haut-Ogooué	320	113	35.3% (30.1–40.8)	0.12	3	0.9% (0.2–2.7)	0.09
Ngounié	199	76	38.2% (31.4–45.3)		4	2.0% (0.6–5.1)	
Nyanga	156	70	44.9% (36.9–53.0)		7	4.5% (1.8–9.0)	
Ogooué-Ivindo	108	34	31.5% (22.9–41.1)		5	4.6% (1.5–10.5)	
Ogooué-Lolo	237	79	33.3% (27.4–39.7)		7	3.0% (1.2–6.0)	
HTLV-1 status							
Non infected	896	333	37.2% (34.0–40.4)	0.22	20	2.2% (1.4–3.4)	0.08
Infected	124	39	31.5% (23.4–40.4)		6	4.8% (1.8–10.2)	
Total	1020	372	36.5% (33.5–39.5)		26	2.5% (1.7–3.7)	

IFA: Immunofluorescence assay; *n*: Total number of tested individuals; *n*IFA: Number of seropositive results at dilution 1:160; *n*PCR: Number of HHV-8 positive PCR on K1 gene.

**Table 2:** HHV-8 PCR results according to serological status at the three dilutions (1:40, 1:80, 1:160).

HHV-8 Serological Results	*n*	HHV-8 Sero-Prevalence (95%CI)	*n* PCR + K1	Percentage of Positive PCR (95%CI)	*p*-Value
IFA 1:40					
Positive	493	48.3% (45.2–51.5)	19	3.9% (2.3–6.0)	
Negative	527		7	1.3% (0.5–2.7)	0.01
IFA 1:80					
Positive	455	44.6% (41.5–47.7)	17	3.7% (2.2–5.9)	
Negative	565		9	1.6% (0.7–3.0)	0.03
IFA 1:160					
Positive	372	36.5% (33.5–39.5)	13	3.5% (1.9–5.9)	
Negative	648		13	2.0% (1.1–3.4)	0.15

*n*: Total number of tested individuals; *n*PCR + K1: Number of individuals with an HHV-8 positive PCR on K1 gene.

## Data Availability

The sequences generated in this study are openly available in GenBank database at reference number MT900801 to MT900831.
